# Cervical Sympathetic Chain Schwannoma in a Child: Anesthesia Concerns

**DOI:** 10.7759/cureus.92750

**Published:** 2025-09-19

**Authors:** Marium Amir, Anum Sohail, Hafiz Sohaib Fraz

**Affiliations:** 1 Anesthesiology, Shaikh Zayed Hospital, Lahore, PAK; 2 Otolaryngology - Head and Neck Surgery, Shaikh Zayed Hospital, Lahore, PAK

**Keywords:** anesthesia concerns in cervical sympathetic chain schwannoma, carotid space schwannoma, cervical sympathetic chain schwannoma, head and neck schwannoma, horner’s syndrome, parapharyngeal space tumor, pediatric cervical sympathetic chain schwannoma, pediatric difficult airway, video-laryngoscope

## Abstract

Cervical sympathetic chain schwannomas are rare benign tumors, extremely uncommon in children, and may present diagnostic and anesthetic challenges due to their proximity to the airway and major vascular structures. We report the case of a 12-year-old boy with a progressively enlarging left-sided cervical mass associated with recent dysphagia and odynophagia. Imaging suggested a vagal schwannoma within the carotid sheath, displacing carotid arteries, internal jugular vein, and trachea, raising concerns for airway distortion and potential hemodynamic instability during surgery. Anticipating a difficult airway and autonomic disturbances, the anesthesia team prepared a comprehensive strategy including a difficult airway cart and readiness for emergency tracheostomy. Standard monitoring was supplemented with invasive arterial blood pressure monitoring. Induction and tracheal intubation were successfully performed using video laryngoscopy, and anesthesia was maintained with careful titration of inhalational agents and neuromuscular blockade. Surgical excision revealed the tumor to originate from the cervical sympathetic chain rather than the vagus nerve. The mass was excised completely with preservation of adjacent nerves and vessels, and the child remained hemodynamically stable throughout the procedure. Recovery was smooth, with no airway compromise, though mild ptosis and miosis consistent with Horner’s syndrome were noted. This case underscores the rarity of pediatric cervical sympathetic chain schwannomas and highlights the need for meticulous anesthetic planning to address airway distortion, vascular involvement, and potential autonomic instability. Early anticipation and multidisciplinary coordination are key to achieving safe surgical and functional outcomes in such rare pediatric head and neck tumors.

## Introduction

Schwannomas are benign, encapsulated tumors that arise from Schwann cells of the peripheral nerve sheath. They account for 20-27% of parapharyngeal space tumors and are most frequently encountered in adults between the second and fourth decades of life [1,2]. Pediatric schwannomas are rare, with fewer than 10% of cases reported in patients under 21 years, and are exceedingly rare in children below 10 years of age [3]. Within this group, cervical sympathetic chain schwannomas are even rarer, with only a few pediatric cases documented in the literature [4].

These tumors usually present as a slow-growing, painless cervical swelling. Mass effect may produce dysphagia, hoarseness, or cranial nerve deficits, while sympathetic chain involvement may lead to Horner’s syndrome [5]. Radiologically, they are typically well-circumscribed, heterogeneous lesions on imaging. Microscopically, schwannomas are encapsulated tumors with Antoni A spindle cell fascicles showing palisading and Verocay bodies, and Antoni B areas that are loose and microcystic [6].

Complete surgical excision is the treatment of choice. However, surgical morbidity can include hoarseness, pharyngo-laryngeal anesthesia, aspiration, and cranial nerve palsy, which may be transient or permanent [7]. From the anesthesiologist’s perspective, large cervical or parapharyngeal masses raise important concerns regarding airway management. Distortion of upper airway anatomy, limited mouth opening, and displacement of the larynx can make tracheal intubation difficult, while sudden airway obstruction may occur on induction. Careful preoperative evaluation, preparedness for awake intubation, and coordination with the surgical team are therefore critical [8].

In our case, a 12-year-old boy presented with a left-sided cervical mass and underwent successful surgical excision. The postoperative course was uneventful, with no evidence of vocal cord paralysis, dysphagia, or aspiration. This case underlines the rarity of sympathetic chain schwannomas in the pediatric age group and highlights that with meticulous surgical technique, excellent functional outcomes can be achieved even in complex head and neck tumors.

## Case presentation

A 12-year-old boy, weighing 40 kg, presented to the ENT department with a left-sided pulsating neck swelling for four years, who was scheduled for cervical peripheral nerve schwannoma excision.

**Figure 1:**
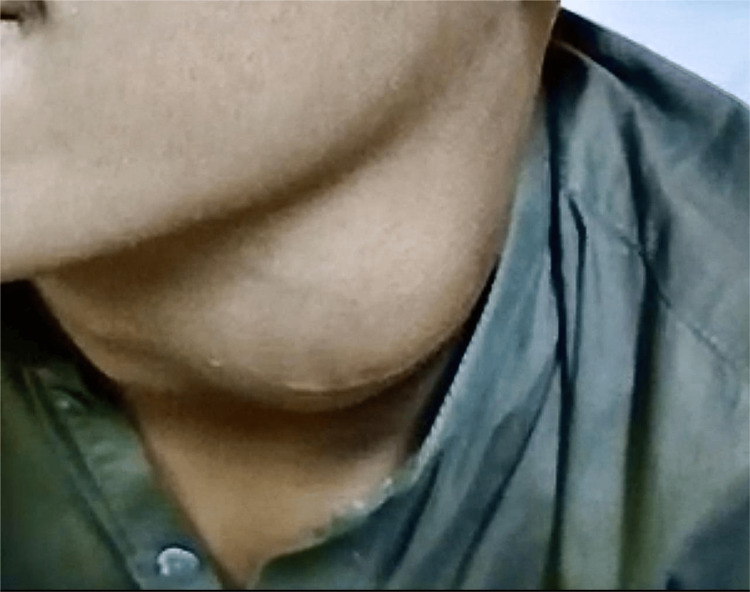
Left-sided swelling with previous scar mark from a biopsy taken.

History revealed that the swelling progressively increased in size over six months. He had complaints of dysphagia and odynophagia with solid foods for the last week. On examination, a single, firm mass extending from the angle of the mandible to the thyroid cartilage. The overlying skin had a previous scar mark from a biopsy taken (Figure 1), and no local rise of temperature. Skin was pinchable, and mass was mobile over vertical and horizontal planes. There were visible pulsations of the carotid vessels superficial to the tumor. The rest of the systemic examination was unremarkable.

**Figure 2:**
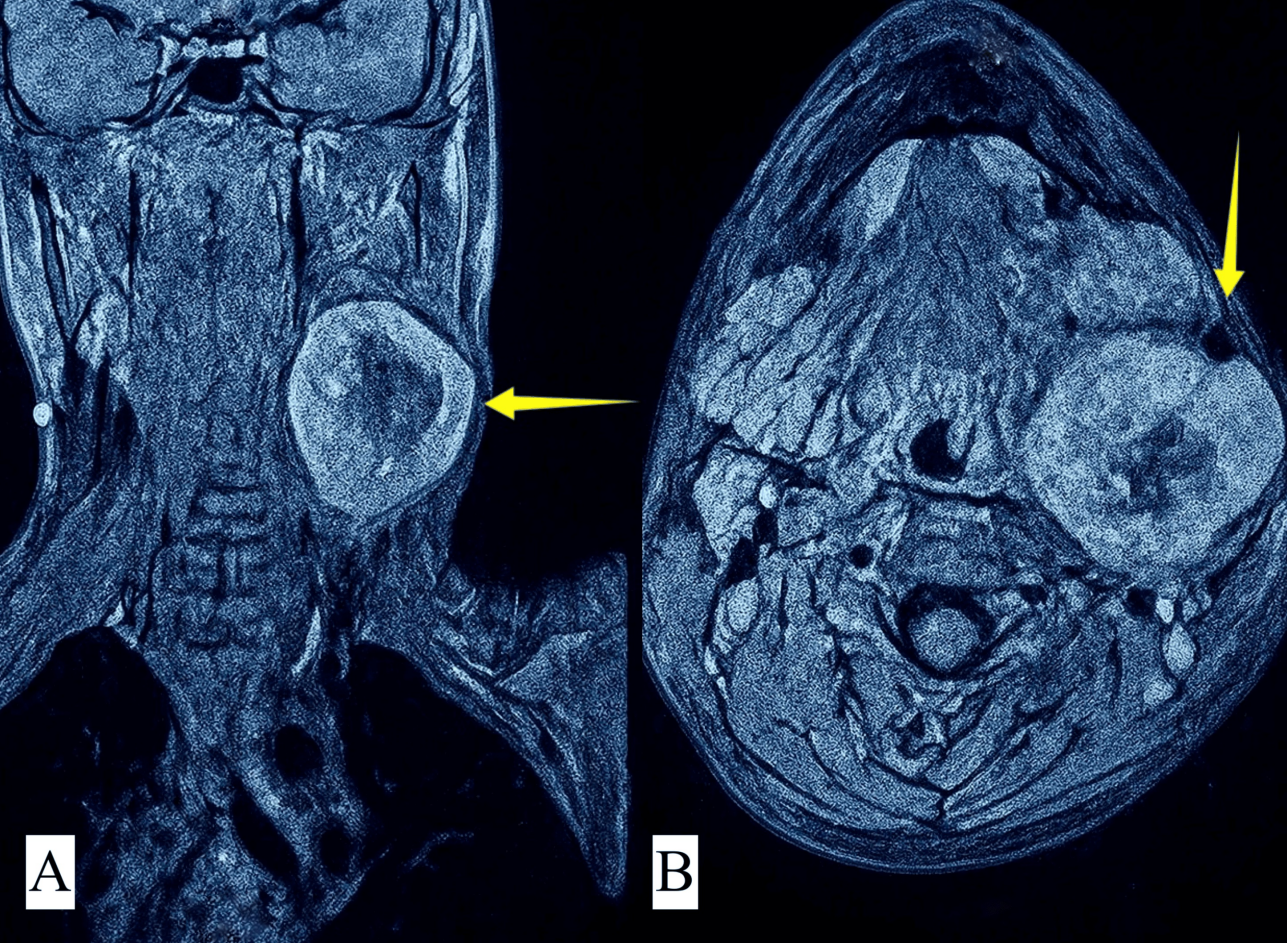
MRI with contrast. A) Parasagittal view. B) Axial view. Arrows showing well-defined heterogeneous lesion in left carotid sheath causing displacement of carotid arteries and internal jugular vein.

Contrast-enhanced computed tomography (CT) of the head and neck with contrast revealed a well-defined fusiform soft tissue density mass in the left carotid sheath from the C3 superior endplate to the inferior endplate of C6, with irregular central hypodensity, and multiple tiny blood vessels were seen. Mass was causing anterior displacement of the common carotid artery. Medially, the mass caused a mild bulge in the pharynx. Magnetic resonance imaging (MRI) of the neck with contrast (Figure 2) revealed a 48 mm × 53 mm × 68 mm lesion in the left carotid sheath with central necrosis and prominent flow voids, causing anterior displacement of the left carotid arteries and carotid bifurcation, with posterior displacement of the internal jugular vein, typically suggestive of vagal schwannoma. All blood investigations, X-ray chest, and electrocardiogram were normal.

On airway assessment, the trachea was deviated toward the opposite side, Mallampati was grade II, and there was a mouth opening of more than three fingers. Difficult intubation was anticipated due to a distorted airway and the possibility of airway collapse on induction.

Informed consent was taken, and the procedure was explained to the parents. The patient was premedicated with oral midazolam the night before surgery and on the day of surgery. The Cardiothoracic surgery team was on board in case of vessel injury and anastomoses. An ICU bed was booked for the untoward consequences of vagus nerve injury. Before anesthesia, a thorough preoperative examination and operating room preparation were completed, including the procurement of a difficult airway cart, video laryngoscope, fiber-optic bronchoscope, laryngeal mask airway (LMA), tracheostomy set, and emergency drugs, including adrenaline, atropine, glycopyrrolate, steroids, and lignocaine IV, to manage hemodynamic collapses.

In the operating room, an 18G and a 20G intravenous cannula were secured. Standard ASA II monitors were attached, including electrocardiography, a non-invasive blood pressure monitor, and pulse oximetry. His pre-induction heart rate was 93 beats per minute, and his blood pressure was 115/72 mmHg. After administering midazolam 2 mg, an inhalational anesthetic of sevoflurane 3% was given to the patient. A direct laryngoscopy was performed using a video laryngoscope, and it revealed Cormack and Lehane grade 2A intubation with vocal cords lateralized to the right side due to mass effect. Then, standard general anesthesia induction was done using nalbuphine 4 mg and propofol 120 mg. Rapid-acting suxamethonium 60 mg was given, and the trachea was intubated using a 6.5 mm ETT with a subglottic suction line. Ventilation was confirmed by bilateral auscultation, and EtCO2 was confirmed using capnography, and the tube was secured. Subsequently, an induction dose of atracurium 20 mg was given after the return of motor function. Anesthesia was maintained using oxygen 2 L/minute, isoflurane 1.5%, and maintenance doses of atracurium. The patient remained hemodynamically stable postinduction. The right radial artery was cannulated for monitoring intra-arterial blood pressure. Dexamethasone 4 mg, paracetamol 400 mg, and hydrocortisone 80 mg were given intraoperatively.

**Figure 3:**
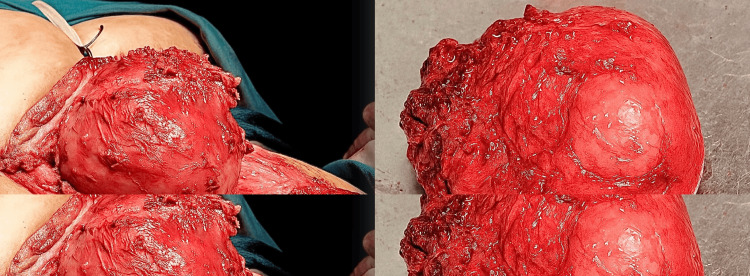
A) Tumor prominently separated from adjacent vascular structures. B) Well-defined tumor resected as a whole.

Surgery lasted for five hours, during which the tumor was found to be arising from the sympathetic chain instead of the vagus nerve, which was excised and removed (Figure 3) as a whole piece; then the biopsy was sent. The hypoglossal nerve, the spinal accessory nerve, and the vagus nerve were identified and saved using careful dissection. The internal jugular vein was saved, but the external jugular vein was ligated. The sympathetic trunk was then repaired, anastomoses were done, and hemostasis was secured.

The patient remained hemodynamically stable throughout the procedure. Neuromuscular blockade was reversed successfully by neostigmine 2.25 mg and glycopyrrolate 0.2 mg at the end of surgery. The left vocal cord was found to be functioning on direct laryngoscopy. Recovery was smooth and uneventful, and the patient was shifted to a high dependency unit for close monitoring and observation. 

**Figure 4:**
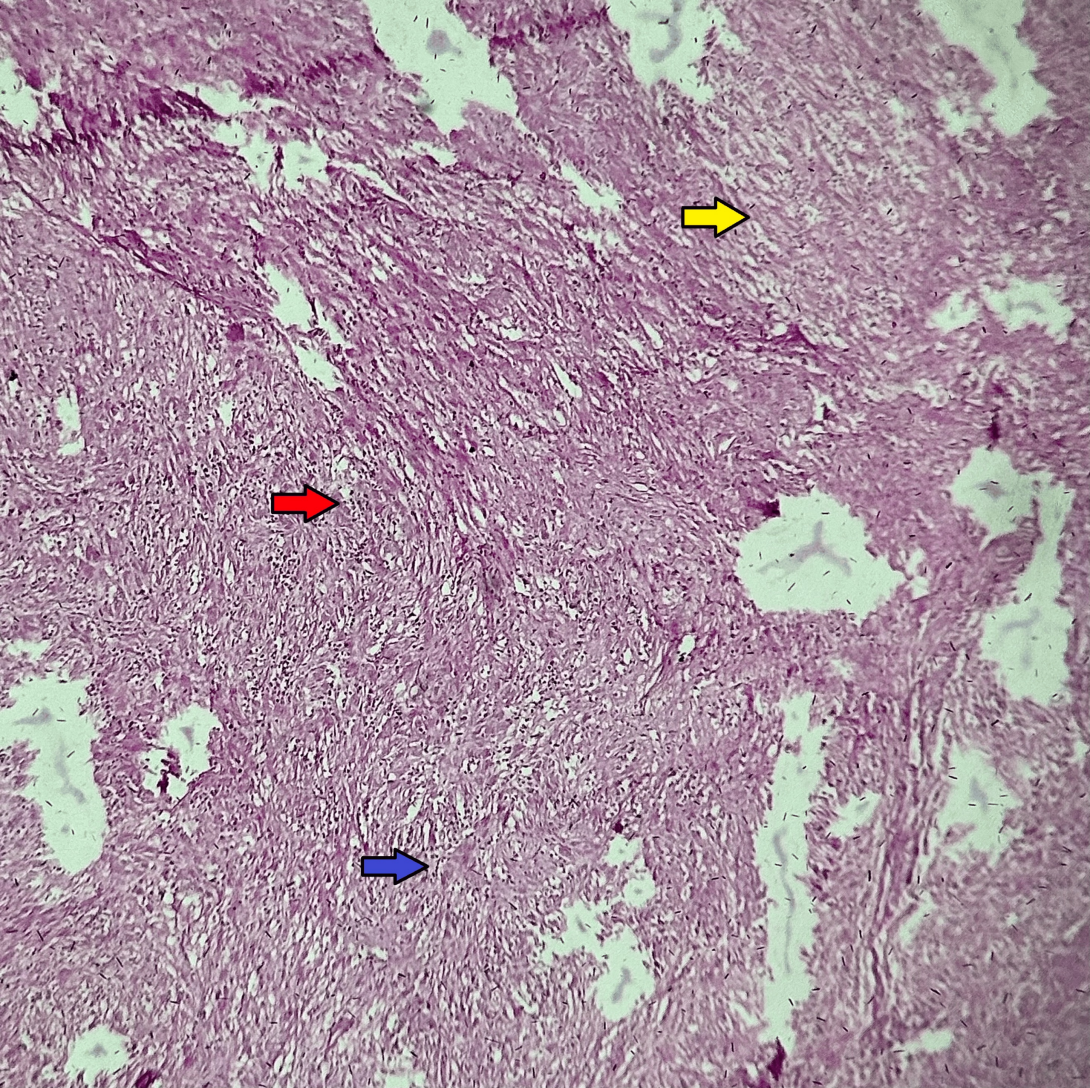
Histological section of the tissue, showing Antoni A areas, Antoni B areas, and Verocay bodies (H&E stain, ×10). Red arrow: Antoni A. Yellow arrow: Antoni B. Blue arrow: Verocay body.

Histopathological report of the specimen (Figure 4) revealed spindle cell lesions with vague hypo and hypercellular sheets of oval cells forming Verocay bodies consistent with the diagnosis of schwannoma. 

Subsequently, the patient developed mild ptosis and slight miosis a week after surgery, but no anhidrosis. The patient was then referred for rehabilitation therapy over the next few months.

## Discussion

Cervical sympathetic chain schwannomas are exceedingly rare in the pediatric population, with only a few cases reported [9-12]. While head and neck schwannomas comprise approximately 25-45% of all extracranial schwannomas, cervical sympathetic chain schwannomas themselves represent less than 5% of these tumors, with pediatric cases being exceedingly rare [4]. Our case matches published reports in terms of clinical presentation: the patient presented with a painless, gradually enlarging lateral neck mass and did not exhibit preoperative Horner’s syndrome, which is typical in pediatric cervical sympathetic chain schwannomas [9,10,12]. As with the case described by Awasthi and Dutta, our patient’s initial presentation did not provide specific neurological symptoms, heightening diagnostic uncertainty [9]. An attempted biopsy prior to referral left a scar but failed to yield a diagnosis, reflecting the literature’s consensus that FNAC and open biopsies often have low diagnostic yield in these tumors due to deep localization and nonspecific cytological findings [9,10,13]. Preoperative imaging in our case revealed a mass with MRI characteristics suggestive of a vagal schwannoma, well-circumscribed, with T1 hypointensity and T2 hyperintensity, mirroring diagnostic challenges noted in earlier reports, where the distinction between vagal and sympathetic chain schwannomas is difficult on imaging alone [10,13,14]. The histopathological report confirmed the diagnosis of schwannoma, showing spindle cells revealing hypercellular and hypocellular areas, which gave rise to Antoni A and B types of patterns and prominent Verocay bodies [6,10]. The definitive diagnosis was established intraoperatively, when the tumor was found to originate from the cervical sympathetic chain rather than the vagus nerve, a scenario frequently described in the literature [10,11]. Surgical excision remains the standard treatment. The anesthetic management of cervical sympathetic chain schwannomas poses distinct complications owing to the tumor’s size and site, and preoperative airway assessment, anticipation of difficult intubation, and the availability of strategies, such as awake fiberoptic or video-assisted laryngoscopy, are critical for patient safety, while maintaining backup options such as tracheostomy [8]. In our case, the patient presented with compressive symptoms, raising concerns about potential airway compromise. Accordingly, a thorough preoperative airway assessment was performed. Consistent with recommendations in the literature, we opted for video laryngoscopy as it provides superior glottic visualization in distorted anatomy and allows for controlled intubation in children with suspected airway difficulty [8,15]. During surgery, manipulating the tumor around might cause serious hemodynamic instability. Pulling or cutting near the sympathetic chain might cause bradycardia, hypotension, or arrhythmias. A case report showed that a patient had acute bradycardia and hypotension during the removal of a cervical sympathetic chain schwannoma, which meant that atropine and vasopressors had to be given right away to stabilize the patient [15]. Thus, the anesthesia team should be ready to handle abrupt changes in the cardiovascular physiology. After surgery, patients should be monitored regularly for problems such as airway compromise, hematoma formation, and neurological impairments. Signs of Horner’s syndrome should be looked for very carefully since they could happen if the sympathetic chain is injured by accident during surgery. This syndrome can cause ptosis, miosis, and anhidrosis on the affected side, but it usually goes away on its own. Also, the danger of aspiration because of possible vocal cord problems should be taken into account, especially if the vagus nerve is affected. To deal with these problems well, the surgical, anesthesia, and critical care teams must plan and work together very carefully. Our patient developed postoperative Horner’s syndrome, characterized by ptosis and miosis on the affected side. This complication is commonly reported and results from the necessary sacrifice of sympathetic fibers during tumor removal [9]. Despite this, our patient experienced no other perioperative or postoperative complications, and the overall outcome was favorable, with no recurrence observed on follow-up [1,9,12]. Given the clinical and radiological overlap with other neck masses, especially vagal schwannoma, this case reinforces the importance of considering cervical sympathetic chain schwannomas in the differential diagnosis of pediatric neck tumors and in managing them intraoperatively by the anesthesia team, particularly when initial investigations are inconclusive [10-12,16].

## Conclusions

Pediatric cervical sympathetic chain schwannomas are extremely uncommon and pose distinctive anesthetic challenges owing to airway displacement, closeness to major blood vessels, and possible autonomic disturbances. Thorough preoperative evaluation, anticipation of a challenging airway, preparedness for intraoperative cardiovascular changes, and coordinated multidisciplinary care are vital to achieve safe tumor removal and optimal postoperative recovery. Even in complex head and neck cases, careful anesthetic and surgical planning can reduce complications and ensure excellent functional outcomes.
